# Small-bowel transection after peroral motorized spiral enteroscopy

**DOI:** 10.1016/j.igie.2023.05.004

**Published:** 2023-07-26

**Authors:** Partha Pal, Pradeep Rebala, Zaheer Nabi, Manu Tandan, D. Nageshwar Reddy

**Affiliations:** Asian Institute of Gastroenterology, Hyderabad, India

A 52-year-old man presented with recurrent small-bowel bleeding. Capsule endoscopy showed a few proximal–mid ileal erosions. Magnetic resonance enterography was unremarkable. The patient’s body mass index was 24.6 kg/m^2^, and he had no history of surgery. He underwent motorized spiral enteroscopy from the antegrade route. Enteroscopy up to the mid–distal ileum was normal, beyond which there was nonprogression. Total enteroscopy was attempted with manual abdominal pressure and position changes but was not successful after considerable attempt and time (nearly 30 minutes). Although there was further progression of the tip of the scope, there was no progression of the part of enteroscope where the spiral overtube is attached on fluoroscopy.

On withdrawal of the scope after putting clips at the depth of maximal insertion (Fig. 1A), there was moderate resistance that was overcome by forward and backward rotation of the overtube with sudden loss of resistance. On further withdrawal, the peritoneal cavity was entered, and the liver was seen (Fig. 1B). On withdrawal out of the peritoneum, a transected end of the bowel was encountered (Fig. 1C). On urgent laparotomy, there was complete transection of the bowel at the mid ileum (Fig. 1D), which was resected and side-to-side anastomosis performed. Intra-operative enteroscopy revealed a distal ileal vascular malformation, which was also resected. On withdrawal of the enteroscope, part of the transected bowel adhered to the proximal part of the overtube where the covering was partially dislodged (Fig. 1E). The patient recovered well after laparotomy and had no further episodes of bleeding at 6 months’ follow-up.

**Figure 1:**
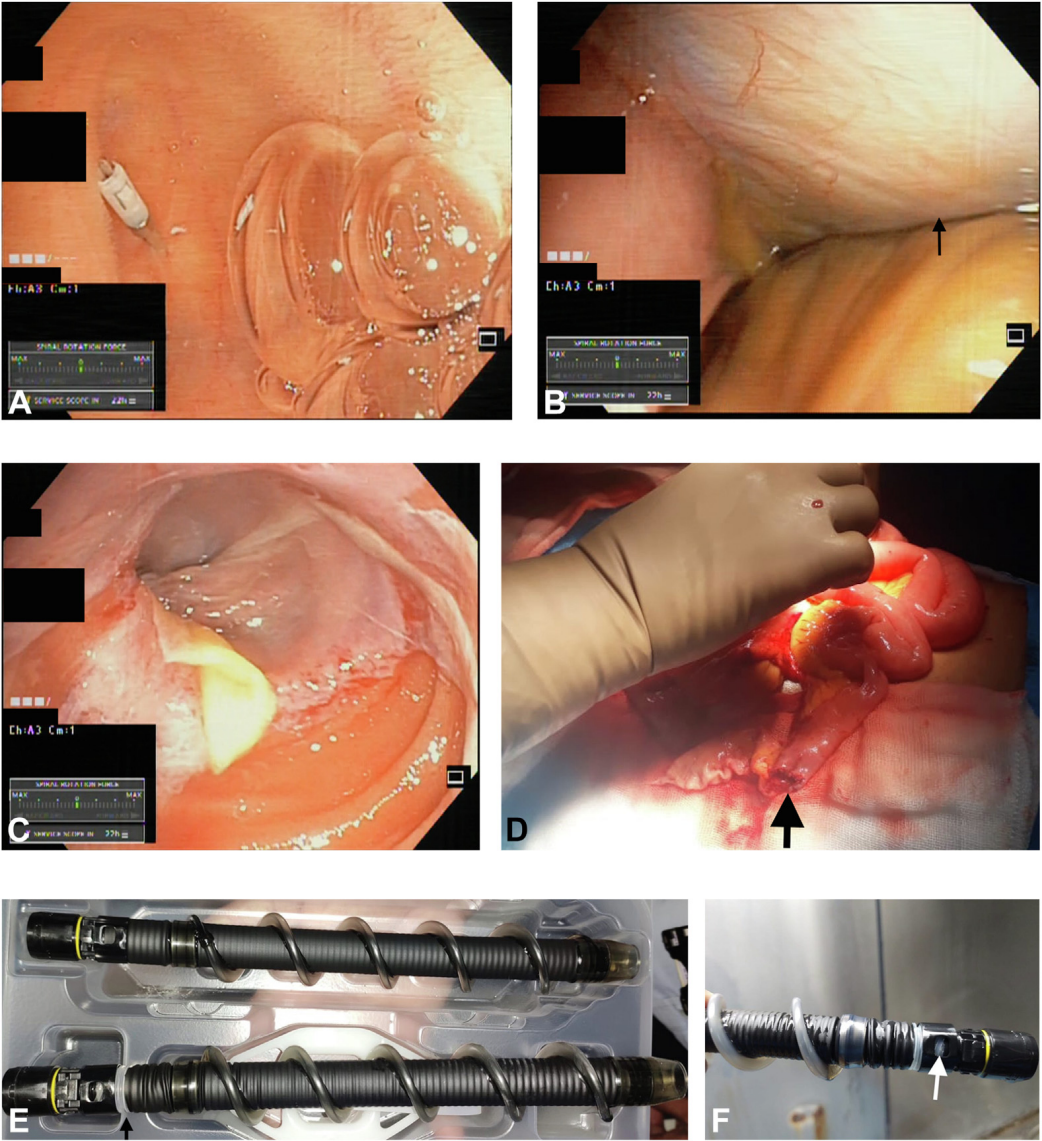
**A**, Clip put at the depth of maximal insertion. **B**, Peritoneal cavity entered on withdrawal of enteroscope (*arrow - liver*). **C**, Lacerated end of the bowel on further withdrawal of enteroscope. **D**, Transected bowel in mid ileum (*arrow*) at laparotomy. **E**, Partially dislodged covering of overtube at proximal part (*below*) compared to normal, unused overtube (*above*). **F**, Detachment of the covering of spiral overtube and displacement of fins from underlying scaffold.

A recent systematic review has suggested that the pooled rate of adverse events with motorized spiral enteroscopy is 17% and that they are mainly minor.^1^ The rate of serious adverse events was only 1%; this included perforation, deep intramural defects, pancreatitis, and significant bleeding requiring transfusion/re-intervention. Gastroesophageal intussusception has been reported with peroral manual spiral enteroscopy with large hiatal hernia as predisposing factor. We speculate that similar intussusception at the segment of the spiral overtube attachment portion led to complete transection of the bowel while attempting withdrawal.^2^ This led to the detachment of the covering of the overtube and displacement of fins from the underlying scaffold (Fig. 1F).

This case highlights the unique adverse event of motorized spiral enteroscopy. Hence, in cases of nonprogression at a given part of the bowel even after change in position and manual abdominal pressure, the scope should be quickly withdrawn without persistent attempt, and enteroscopy should be done from the opposite route if the desired lesion is not reached.

## Disclosure

All authors disclosed no financial relationships.
